# Direct *In Vivo* Cell Lineage Analysis in the Retrorsine and 2AAF Models of Liver Injury after Genetic Labeling in Adult and Newborn Rats

**DOI:** 10.1371/journal.pone.0007267

**Published:** 2009-09-30

**Authors:** Virginie Pichard, Dominique Aubert, Nicolas Ferry

**Affiliations:** INSERM U948, Biothérapies Hépatiques, CHU Hotel Dieu, Nantes, France; Stockholm University, Sweden

## Abstract

**Backgrounds and Aims:**

When hepatocyte proliferation is impaired, liver regeneration proceeds from the division of non parenchymal hepatocyte progenitors. Oval cells and Small Hepatocyte-like Progenitor Cells (SHPCs) represent the two most studied examples of such epithelial cells with putative stem cell capacity. In the present study we wished to compare the origin of SHPCs proliferating after retrorsine administration to the one of oval cells observed after 2-Acetyl-Amino fluorene (2-AAF) treatment.

**Methodology/Principal Findings:**

We used retroviral-mediated nlslacZ genetic labeling of dividing cells to study the fate of cells in the liver. Labeling was performed either in adult rats before treatment or in newborn animals. Labeled cells were identified and characterised by immunohistochemistry. In adult-labeled animals, labeling was restricted to mature hepatocytes. Retrorsine treatment did not modify the overall number of labeled cells in the liver whereas after 2-AAF administration unlabeled oval cells were recorded and the total number of labeled cells decreased significantly. When labeling was performed in newborn rats, results after retrorsine administration were identical to those obtained in adult-labeled rats. In contrast, in the 2-AAF regimen numerous labeled oval cells were present and were able to generate new labeled hepatocytes. Furthermore, we also observed labeled biliary tracts in 2-AAF treated rats.

**Conclusions:**

Our results srongly suggest that SHPCs are derived from hepatocytes and we confirm that SHPCs and oval cells do not share the same origin. We also show that hepatic progenitors are labeled in newborn rats suggesting future directions for in vivo lineage studies.

## Introduction

Hepatic regeneration after liver injury is normally accounted for by division of mature hepatocytes. However, when hepatocyte division is impaired, proliferation of non parenchymal cells occurs to eventually replace the injured parenchyma. A large variety of animal models have been used to study the origin and the role of these non-parenchymal liver stem cells and one constant finding from these studies was the wide heterogeneity of the stem cell populations involved. Two main categories of putative hepatic progenitors have been described which differ morphologically and phenotypically: oval cells (according to the oval shape of their nucleus) and SHPCs (for Small Hepatocyte-like Progenitor Cells) [1]. Other populations of putative stem cells have been described but their actual existence is dubious, mostly because the search for specific markers of hepatic progenitor cells has not yet been completely successful. Indeed, most of the initially described markers of oval cells are actually expressed by biliary cells, fetal hepatocytes or even SHPCs. More recently-described markers also pointed out to the mixed epithelial/mesenchymal phenotype of oval cells [2]. The search for specific markers is a critical issue since most of the knowledge regarding the origin and the fate of liver stem cells is based on such phenotypic characterisation with the assumption that cells expressing the same markers pattern may have a common origin. As an example, a number of hematopoietic markers were described in various models of oval cell proliferation (including thy-1, CD34, Sca1) supporting the hypothesis that oval cells might derived from bone marrow [3], [4]. On the opposite, recent studies revealed that hematopoietic markers may be not expressed in the true stem cell fraction of the oval cell population [5], [6] thus indicating that oval cells are not from bone marrow origin but may originate either from the canal of Hering as suspected long ago [7], [8] or from other hepatic stem cell niches identified inside the liver [9]. The origin of SHPCs is also controversial. Many previous lineage studies in the dipin as well as in the retrorsine model of hepatic injury support the notion that SHPCs derive from hepatocytes that escape the effect of toxic compound [10], [11], [12]. However, it has also been suggested that SHPCs originate from a new stem cell population or even from oval cells [13], [14]. However the latter hypothesis was refuted by subsequent studies [15], [16].

In the present study, we wished to use in vivo genetic labeling to trace the fate of specific cell populations in the liver after liver intoxication with two different compounds: retrorsine which is known to induce proliferation of SHPCs and 2-Acetyl aminofluoren (2-AAF) which results in the proliferation of oval cells. In adult animals, mature hepatocytes were specifically labeled and their lineage was analyzed in response to either drugs. We also labeled cells with lacZ recombinant retroviral vectors injected at 2 days of age in newborn animals that were subsequently treated with either of the two drugs.

## Results

In a first set of experiments we analysed the fate of hepatocyte in the retrorsine and 2-AAF models of liver injury after genetic labeling of hepatocytes in adult animals. Hepatocytes were labeled with recombinant Moloney Murine Leukemia Virus (MoMuLV) vectors carrying the nls lacZ gene after a short treatment with cyproterone acetate (CPA) and triiodothyronin (T3) as previously described [12], [17]. This protocol will force hepatocytes in the cell cycle to render them infectable with MoMULV vectors [18]. To circumvent any cytotoxic immune response to β-galactosidase and allow long term transgene expression, animals received a single dose of a recombinant adenoviral vector carrying a modified CTLA4Ig as previously described. This treatment is known to suppress immune response against β**–**galactosidase for at least 18 weeks in most of the animals with no alteration of liver physiology [19].

**Figure 1:**
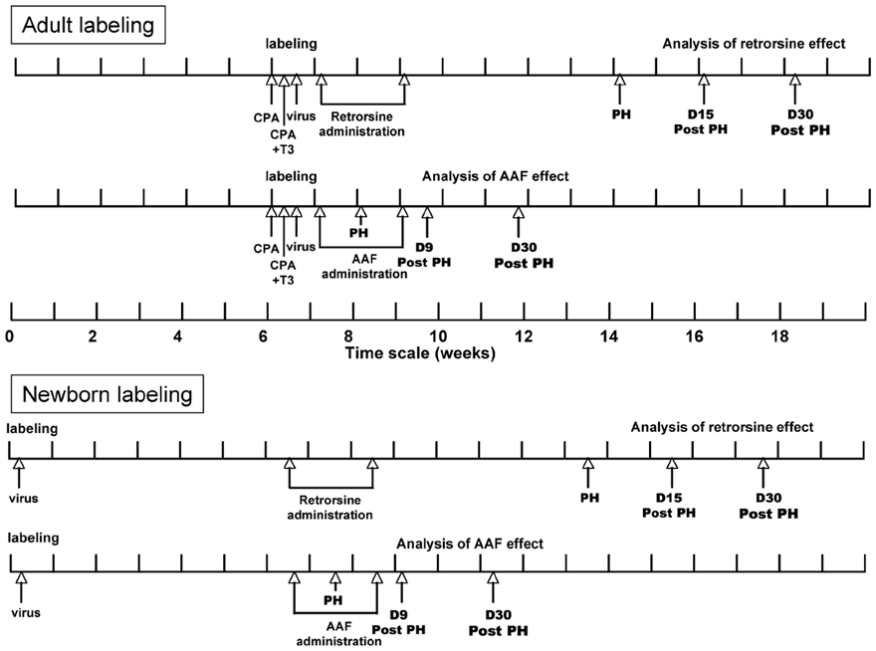
Schematic diagrams of retrorsine and 2-AAF treatments.

After labeling, animals were divided in two cohorts and received retrorsine (n = 10) or 2-AAF (n = 9) according to the schedule depicted in Figure 1. Liver specimens were harvested at the time of hepatectomy as well as at sacrifice in all animals.

**Figure 2:**
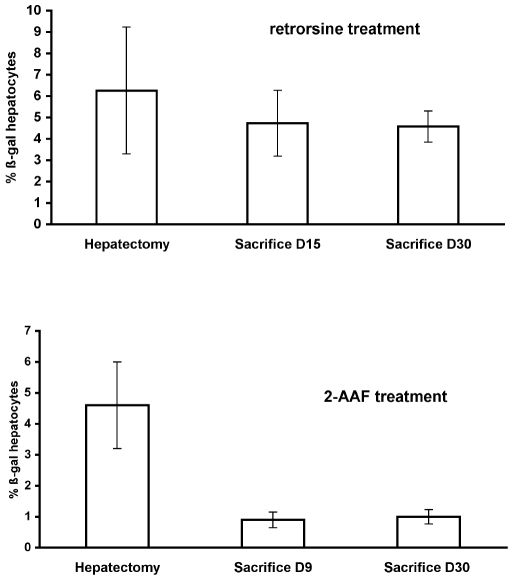
Quantification of the number of β-galactosidase positive hepatocytes in the liver of rat labeled at adulthood and treated with Retrorsine (upper panel) or 2-AAF (lower panel).

**Figure 3:**
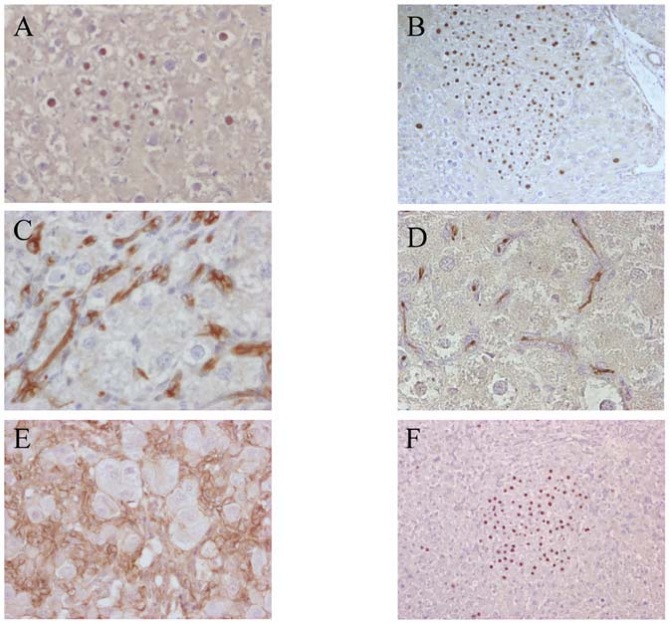
Histological analysis of retrorsin and 2-AAF treated rat liver after genetic labeling of mature hepatocytes in adult animals. Retrorsin model. (A) β-galactosidase positive cells gathered in clusters of small hepatocyte day 15. (B) same clusters at day 30 post hepatectomy. 2-AAF model. Immunohistochemical detection of CKs (C), GGT (D), and β-catenin (E) expressed by oval cells. (F) Clusters of β-galactosidase positive hepatocyte observed in the 2-AAF model. All sections are hematoxylin counterstained. All magnifications: ×400 except ×200 B and F.

In the retrorsine group, the liver lobes removed at partial hepatectomy revealed a mean proportion of 6.3±3% of β-galactosidase positive hepatocytes (Table 1). This value is in the same range than in animals that did not receive retrorsine [11], [12]. We also observed, that cells other than hepatocytes were not labeled (<0.01%) as previously described [12]. The labeled cells were randomly distributed in the liver lobule, indicating indiscriminate infection of hepatocytes with the retroviral vector [11], [20], [21]. In two animals (R4 and R6 in Table 1), no β-galactosidase positive hepatocytes were found. However, anti β-galactosidase antibodies were found in the serum indicating that some animals may escape immune suppression induced by CTLA4Ig. Four animals were sacrificed at day 15 after hepatectomy. In these animals the mean proportion of β-galactosidase positive hepatocytes was 4.7±1.5% (Figure 2). Moreover, we observed the presence of clusters of small-size hepatocytes (less than 20 cells in size) which corresponded to Small Hepatic Progenitor cells (SHPCs) as previously described [10]. These clusters were highly labeled with anti Ki67 antibody indicating a high proliferation index. As shown in Figure 3A some clusters also expressed β-galactosidase and the proportion of clusters expressing β-galactosidase was similar to the proportion of hepatocytes that were initially labeled (Table 1). Four animals were sacrificed at 4 weeks after hepatectomy. In these animals, we found larger clusters of small hepatocytes, resulting from the division of SHPCs as expected in the retrorsine model at this time point [10], [11]. Moreover, at that time the Ki67 labeling was much lower, indicating a drop in cell proliferation in the clusters. We also observed that some clusters expressed β-galactosidase (Figure 3B). Because these large clusters frequently merged, precise quantification of the proportion of β-glactosidse positive clusters was not possible. However, the mean overall proportion of β–gal positive hepatocytes was 4.6±0.7%. We never detected clear oval cell proliferation in any section at sacrifice although transient ductular reaction was sometimes seen. Cytokeratin staining was limited to biliary epithelial cells. These data recapitulated our previous results and showed that the mean proportion of β-galactosidase positive hepatocytes was not statistically different in livers harvested at hepatectomy or at the two times of sacrifice (p = 0.4 using ANOVA). Moreover, the proportion of β-galactosidase clusters at day 15 was identical to the proportion of labeled hepatocytes, strongly suggesting that the clusters originated from the labeled hepatocytes that were present in the liver before retrorsine administration. To confirm that the presence of merging clusters of various sizes did not impact the counting of hepatocytes, we verified that β-galactosidase enzymatic activity was identical in liver homogenates from rats sacrificed at day 15 or day 30 (17.9±6.1 ng βgal/mg protein at day 15 vs 18.5±5.6 at day 30; p = 0.9 using Student's *t* test).

**Table 1:** Quantification of β-galactosidase positive hepatocytes and clones in the liver of adult-labeled rats treated with retrorsine.

	%βgal hepatocytes	%βgal hepatocytes	%βgal hepatocytes	% βgal clones
rat number	**hepatectomy**	**day 15**	**day 30**	**day 15**
R1	11	7		10.1
R2	10.1	4.3		3.2
R3	7.2	4		5.7
R4	0			
R5	4.3	3.6		4.6
R6	0			
R7	6		4	
R8	4.5		5.6	
R9	4.4		4.6	
R10	2.6		4.1	
**mean**	**6.3±3**	**4.7±1.5**	**4.6±0.7**	**5.9±2.9**

In the 2-AAF cohort (n = 9), the mean proportion of β-galactosidase positive cells at the time of hepatectomy was 4.5±1.36% (Figure 2) similar to the one observed in the retrorsine group at hepatectomy (p = 0.23 using Student's *t* test). One animal died immediately after hepatectomy from unknown cause. In the liver of rats sacrificed at day 9 post hepatectomy (n = 4) immunohistochemistry revealed 0.9±0.25% of β-galactosidase positive hepatocytes. Oval cells were detected in large amounts and were positive for Gamma Glutamyl Transpeptide (GGT) and β-catenin. They were also labeled with a panel of anti cytokeratins antibodies which do not label hepatocytes but are known to detect oval cells with a sensitivity and specificity greater that 99% (Figure 3C–E) [22]. We never detected the presence of oval cells that were positive for β-galactosidase. Four animals were sacrificed one month after partial hepatectomy. In these animals the overall proportion of β-galactosidase hepatocytes was 1±0.23%. Oval cells were still present at that time, although in lower amounts, but they were consistently negative for β-galactosidase expression. It is noteworthy that in this cohort, we observed a significant decrease in the number of β-galactosidase positive hepatocytes between hepatectomy and sacrifice (p = 0.01 using ANOVA). Lower enzyme activity was also recorded at sacrifice (6.9±2.6 ng β-galactosidase/mg prot at day 9 vs 7.45±1.9 at day 30) (p = 0.7 using Student's t test). We also observed in the liver of animals at sacrifice the presence of rare clusters of mature hepatocytes (from 2 to 10 clones/cm^2^). These clusters were always larger than 20 cells (17% from 20 to 50 cells; 59% from 50 to 100 cells and 24% more than 100 cells) (Figure 3F). We already observed such β-galactosidase positive clusters after 2-AAF treatment and we hypothesized that they originated from the division of hepatocytes resistant to the toxic effect of 2-AAF [23]. We cannot completely exclude that some of them arise from labeling of rare progenitor cells because the proportion of these clusters was very low (less than 1 for 2000 cells).

Overall, our data clearly established that after retrorsine treatment the proportion of labeled hepatocytes was stable with the presence of numerous small clones of labeled hepatocytes. In contrast, following 2-AAF treatment the proportion of labeled cells dropped and only rare large clusters of labeled hepatocytes were detected. This indicated that liver regeneration proceeds from different populations in retrorsin treated and 2-AAF treated adult animals. After AAF intoxication, regeneration is known to proceed from the division of oval cells that proliferate and gradually differentiate into hepatocytes [1], [24], [25]. Thus in our model, genetically-labeled hepatocytes were diluted by the proliferation of non-labeled oval cells that proliferated and differentiated in non-labeled hepatocytes.

In a second set of experiments we genetically labeled liver cells by administration of recombinant nls LacZ MoMuLV vectors to newborn rats. The proportion of labeled cells was highly variable between animals ranging from 0.2% to 8% when assessed at different time points.In these animals, we observed the presence of a small proportion of non-hepatocyte cells that were labeled. These cells were mainly in the portal tract represented 0.60% of all labeled cells at day 15, 1.98% at 4 weeks and 1.3% at 7 weeks. After labeling, animals were divided in two groups and received either retrorsine or 2-AAF at six weeks of age (Figure 1).

**Figure 4:**
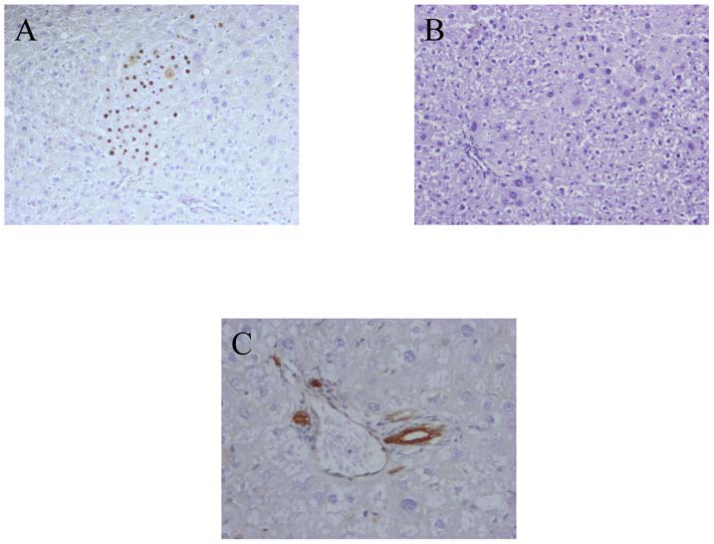
Histological analysis of the retrorsin model after genetic labeling of liver cells in newborn rats. Retrorsin model. (A) Cluster of SHPCs positive for β-galactosidase. (B) Hematoxylin and eosin staining showing large number of merging clusters 30 days post hepatectomy. (C) Immunohistochemical detection of CKs day 30 post hepatectomy. Oval cells are not detected. Hematoxylin counterstain. Magnification: ×200 A–B: ×400 C.

In the retrorsine group (n = 7) the proportion of β-galactosidase positive hepatocytes was evaluated in the liver lobes removed at partial hepatectomy. The mean proportion of β-galactosidase hepatocytes was 1.57±1% (Table 2). In the labeled cells population, 1.83% of the cells were neither hepatocytes nor oval cellsThe presence of β-galactosidase positive cells and the enzyme activity were analysed at sacrifice in the same animals. We observed a robust so-called “SHPC response” with the presence of numerous clusters of proliferating hepatocytes, either isolated or merging in large patches, some of which were also positive for β-galactosidase (Figure 4A–B). As shown in Table 2 there were slightly more β-galactosidase positive hepatocytes at sacrifice (2.07±1.4%) than at the time of hepatectomy. However this difference was not statistically significant (p = 0.79 by Student's *t* test) At the time of sacrifice it was not possible to quantify the proportion of non-hepatocyte labeled cells. It is noteworthy that we never detected oval cells in any section either by morphological examination or by immunohistological staining (Figure 4C). This confirm that SHPCs do not arise from oval cells. Although we never detected a clear proliferation of other non parenchymal cells, we cannot exclude in this setting that some labeled hepatocytes derived from labeled progenitors.

**Table 2:** % β-galactosidase positive hepatocytes in the liver of rats labeled at birth.

treatment	hepatectomy	sacrifice	ratio sacr/hep
retrorsine	0.5	1	2
retrorsine	0.5	0.7	1.4
retrorsine	1.4	2.2	1.57
retrorsine	1.7	1.4	0.82
retrorsine	3.4	4.4	1.29
retrorsine	2.2	3.6	1.63
retrorsine	1.3	1.2	0.92
**mean**	**1.57±1**	**2.07±1.4**	**1.36±0.41**
2-AAF	5.2	8.6	1.65
2-AAF	0.4	0.6	1.5
2-AAF	4	4.9	1.23
2-AAF	0.2	0.5	2.5
**mean**	**2.47±2.5**	**3.65±4.64**	**1.72±0.54**

**Figure 5:**
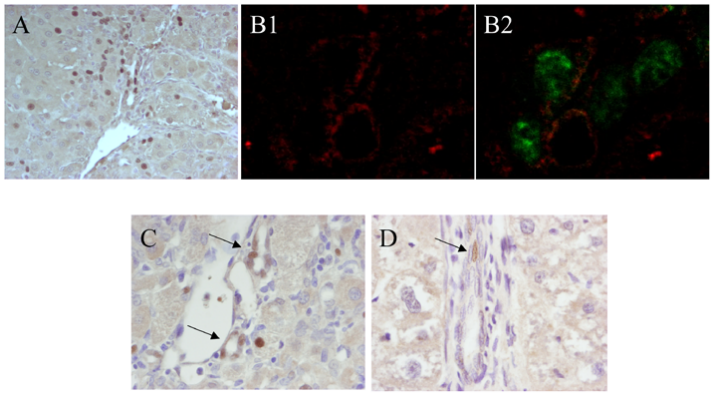
Histological analysis of 2-AAF model after genetic labeling of liver cells in newborn rats. (A) Detection of β-galactosidase positives oval cells. (B1) Immunofluorescent staining of CK in oval cells appearing in red (B2) colocalisation of CK with β-galactosidase appearing in green. (C) Detection of β-galactosidase positive cells in the biliary cell population (arrows). (D) Detection of a positive cells (arrow) near the Hering canal. Hematoxylin counterstain for A, C, D, Magnification: ×400 except for B: ×1000.

In the 2-AAF cohort (n = 4), the proportion of β-galactosidase positive hepatocytes was also highly variable at hepatectomy. However, in sharp contrast to the 2-AAF treated adult cohort, there was no decrease in the proportion of β-galactosidase hepatocytes between hepatectomy and sacrifice (p = 0.63 using the Student's *t* test) (Table 2). We observed a high proportion of β-galactosidase positive oval cells. (Figure 5A). β-galactosidase expression in oval cells was confirmed by β-galactosidase and CKs immuno colocalization coupled with confocal microscopy (Fig. 5 B1–B2). The β-galactosidase positive oval cells were mainly located around the portal tract, in the vicinity of the Hering canal, suggesting that they may arise from transitional cells. The absence of decrease in the number of β-galactosidase hepatocytes in these animals in which a proliferation of labeled oval cells occurred, strongly suggested a lineage relationship between the two populations with labeled oval cells giving rise to labeled hepatocytes. Along the same line, β-galactosidase positive hepatocytes were frequently adjacent to oval cells even at day 9 after hepatectomy (Figure 5A) suggesting that oval cell differentiation in hepatocytes is a rapid process. Another salient observation was the presence of β-galactosidase positive cells in the biliary cell population (Figure 5C). These positive biliary ducts were not frequent and present in 2% of the portal space analysed.

The presence of β-galactosidase positive oval cells and biliary cells prompted us to analyse liver sections in a cohort of newborn injected animals (n = 5) sacrificed at 4 weeks of age (i.e. before any treatment) to look for the presence of putative oval cell precursors. In each animal, 37 to 75 portal spaces were carefully examined. The mean proportion of portal space in which a single positive cell was observed was 12±6.5%. In these portal spaces, the positive cells were most often in zone 1 and 3 of the portal space according to the classification of Petersen and Shupe (Figure 5D) [26]. Only two cells in zone 2 were observed indicating that biliary cells were almost never observed.

## Discussion

Genetic labeling with integrative gene transfer vectors is a powerful approach to study cell lineage in various organs. In the liver, the opportunity to label cells in vivo makes lineage study feasible in the absence of any ex vivo manipulation of the cells that will be studied. In the present study, we wished to use genetic labeling with the nlsLacZ gene to compare the fate of mature hepatocytes in two models of liver injury in which replication of hepatocytes is impaired.

First we present evidence for the different cellular origin of SHPCs and oval cells. SHPCs share antigenic characteristics with hepatocytes and fetal hepatoblasts [14] but their origin has long been debated. They may arise from a distinct population of stem cells present in the liver [14], [27]. Alternatively, it has been suggested that SHPCs are derived from oval cells [13]. However these results were not confirmed [15], [16]. We previously presented evidence that SHPCs are derived from mature hepatocytes [11], [12]. This hypothesis was also previously proposed in dipin-induced toxic injury in mice and hepatocytes resistant to dipin were shown to contribute to the regeneration process [10]. Our present results show that when only hepatocytes are labeled in the adult animals, the number of SHPC clusters expressing the label is identical to the number of labeled hepatocytes before treatement. Moreover, there is no variation in the overall proportion of β-galactosidase hepatocytes after retrorsine administration. These findings strengthen the hypothesis that “SHPCs” are the progeny of hepatocytes as we previouslly suggested [11]. In contrast, after 2-AAF administration in adult labeled animals the number of labeled cells drops significantly. This confirms that oval cells do not arise from hepatocytes and do not share a common origin with SHPCs. Although we cannot formally exclude the possibility that putative stem cells are labeled and give rise to SHPCs, this seems unlikely when labeling is performed in adult rats because: i) when labeling is performed in the adult rat, hepatocytes account for more than 99.9% of labeled cells using our protocol, ii) if non hepatocytes progenitors would be activated after administration of retrorsine, they should not be labeled by MoMULV vectors injected long before retrorsine administration.

Second, we observed a clear difference between animals labeled at adulthood or after birth. In newborn-labeled animals a significant proportion of non parenchymal cells was labeled. This precluded to draw any conclusion regarding the origin of mature hepatocytes in the retrorsine modelAfter 2-AAF injury many oval cells were labeled. In these animals the proportion of β-galactosidase positive hepatocytes was stable in contrast to the results obtained in adult-labeled animals indicating that oval cells were able to generate hepatocytesin this setting. To our knowledge this represents the first report of in vivo genetic labeling of oval cells. Numerous studies have already demonstrated that oval cells isolated from a donor liver and enriched with various systems can give rise to hepatocytes after transplantation. Direct in vivo studies of the fate of oval cells have been previously carried out using ^3^H-thymidine as a marker more that 20 years ago. Tatematsu et al. failed to get evidence for the conversion of oval cells to hepatocytes after ^3^H-thymidine labeling of oval cells [28]. In contrast, Evarts et al. observed transfer of radio-labeled thymidine from oval cells to hepatocytes and suggested a precursor-product relationship between oval cells and hepatocytes [25], [29]. It is worth noting that in this study differentiation of oval cells was rapid and 91% of basophilic hepatocytes contained the label 9 days after partial hepatectomy. Very low labeling was observed in acidophilic hepatocytes that are hepatocytes not originating from oval cells. In the present study we observed numerous labeled hepatocytes in the vicinity of areas of β-galactosidase positive oval cells as early as 9 days after partial hepatectomy. β-galactosidase positive hepatocytes were intermingled with clusters of β-galactosidase positive oval cells a situation compatible with a lineage relationship.

Because labeling occurred six weeks before 2-AAF administration, oval cells were not directly infected with MoMULV vectors. Therefore it is likely that in the newborn, viral vectors were able to infect hepatocytes as well as other precursor cells that were still dividing at the time of infection. These labeled precursors were thereafter detected in the portal spaces of the animals and may include Hering cells. When 2-AAF was given to these animals, the labeled precursors may have been activated to proliferate and give rise to labeled oval cells. We cannot exclude that some other precursor cells different from oval cells can be activated after retrorsine administration and contribute to the regeneration in this setting.. Similarly, the labeled precursors are a likely source of labeled biliary cells that we observed after 2-AAF treatment. However, another possibility is that labeled oval cells may also differentiate in vivo into biliary epithelial cells. This would explain why no labeled biliary cells were detected in the retrorsine model. The bipotential capacity of oval cells has been already suggested [1]. Oval cells are supposed to proliferate and form ductular structures that communicate with the biliray system [30]. To our knowledge, no direct in vivo lineage analysis has yet reported the generation of biliary cells from oval cells in the liver and our data are the first indication that such lineage analysis is feasible.

In conclusion our results demonstrate the validity of genetic labeling to study cell fate in pathological conditions and support the use of this strategy in future studies of hepatic regeneration.

## Materials and Methods

### Animals

Male Fischer 344 rats were used in this study. They were housed at the Nantes School of Medicine animal facility. All surgical procedures were conducted according to the guidelines of the French Ministère de l′Agriculture. Rats were anesthetized by isofluorane inhalation (3% v/v).

Two-thirds hepatectomy was performed according to [31].

Cyproterone acetate (CPA) was administered by force feeding two consecutive days at a daily dose of 200 mg/kg. Triiodothyronine (T3) was administered intra-peritoneally the second day (day 2) at a dose of 4 mg/kg. Two injections of retrovirus (i.e 3×10^9^ infectious units/kg) were performed in the dorsal penile vein [17]. In newborn animals, viral vectors were injected via the temporal vein as described previously (1×10^8^ infectious units/rats) [32].

Retrorsine was added to distilled water at 10 mg/ml and titrated to pH 2.5 with 1N HCL to achieve dissolution. Subsequently, the solution was neutralized using 1 N NaOH, and Nacl was added to a final concentration of 6 mg/ml retrorsine and 0.15 mol/L NaCL (pH 7). Retrorsine was adminstered by intra peritoneal injection. 2-AAF was incorporated at 0.02% in the pellet chow.

### Viral vector

We used amphotropic MoMULV recombinant retroviral vectors containing the E.coli nlsLcZ gene encoding β-galactosidase gene coupled to the nuclear localization signal from the SV40 large T antigene. The viral supernatant was prepared as previously described [20] and the titer was routinely 10^9^ infectious units/ml.

CTLA4-Ig recombinant adenoviral vectors were prepared by the Vector Core of Nantes Hospital as previously described [19]. They harbor the sequence encoding the extracellular portion of the murine CTLA4 fused to human IgG1 Fc fragment, placed under the transcriptional control of the CMV promoter. Recombinant CTLA4Ig adenoviral vectors were injected in the tibialis anterior at a dose of 10^10^ infectious particles/animal immediately after injection of retroviral vectors.

### Histology

The following antibodies were used on formalin-fixed/paraffin-embedded sections (5 µm): Cytokeratines (rabbit anti-cow cytokeratine polyclonal ref Z062201 Dako, France 1∶750), β-galactosidase (1∶1000), gamma glutamyl transpeptidase (GGT, gift from Pr Laperche 1∶150), β-catenin, (1/50), Mib5 antibody (anti Ki67 antibody, 1∶50). Citrate buffer antigen retrieval was used for cytokeratines Mib5 and β-catenin. Primary antibodies were incubated overnight at 4°C revealed with biotinylated goat anti-mouse immunoglobulin and streptavidin-peroxidase using DAB as a chromogenic substrate. Positive cells were counted on at least 10 fields at 40× magnification, corresponding approximately to 3000 hepatocytes. For colocalization of cytokeratin and β-galactosidase, anti CKs antibodies were incubated overnight at 4°C. After washing, sections were incubated with Cy3-conjugated anti–rabbit Ig (1/500) for 30 minutes. Anti β-galactosidase antibodies were then applied for 2 hours and revealed with FITC-conjugated anti-rabbit Ig for 30 minutes. Slides were visualized using Leica SP1 and image processing used Metamorph and ImageJ software.

### β-galactosidase Assay

Beta-galactosidase activity was detected by fluorescence assy using methyllumbelliferyl-β-D-galactoside as previously described [12]. Fluorescence was measured on a fluorometer, with excitation and emission wavelengths of 365 nm and 460 nm respectively.
